# Anti-RSV prophylaxis efficacy for infants and young children with cystic fibrosis in Ireland

**DOI:** 10.1186/s40248-015-0029-9

**Published:** 2015-10-15

**Authors:** Barry Linnane, Miranda G. Kiernan, Nuala H. O’Connell, Linda Kearse, Colum P. Dunne

**Affiliations:** Cystic Fibrosis Unit, University Hospital, Limerick, Ireland; National Children’s Research Centre, Crumlin, Dublin, Ireland; Graduate Entry Medical School and Centre for Interventions in Infection, Inflammation & Immunity (4i), University of Limerick, Limerick, Ireland; Study of Host Immunity and Early Lung Disease in CF (SHIELD CF), Dublin, Ireland; Department of Clinical Microbiology, University Hospital Limerick, Limerick, Ireland

**Keywords:** Paediatric, Cystic fibrosis, RSV, Palivizumab, Efficacy

## Abstract

**Rationale:**

There is limited evidence supporting the routine use of palivizumab in paediatric cystic fibrosis (CF) patients to reduce respiratory syncytial virus (RSV) infection and related hospitalisation. Despite this, anti-RSV prophylaxis is increasingly common. This is the first report from Ireland regarding palivizumab outcomes for children with CF, under 2 years old, despite the greatest prevalence of CF globally.

**Methods:**

An audit was performed at a tertiary hospital in Ireland’s mid-West to document all children with CF, <24 months old, who received palivizumab over a five year period and comparision made with all eligible children for the prior five year period who had not received the product (also CF patients). Palivizumab was administered to both cohorts in their first year of life. Hospitalisation rates were compared using Fisher’s exact test. Incidence of RSV and *Pseudomonas aeruginosa* infection was recorded.

**Results:**

A total of 19 patients who received palivizumab were included in the study; comparision was made with a retrospective control group of 30 patients. Prophylactic palivizumab did not prevent hospitalisation for 10/19 patients, 3 of whom were affected by RSV. This was significantly greater than in the control group, where no hospitalisations were recorded (*p* < 0.0001). *P. aeruginosa* was isolated in one case from the study cohort, while no *P. aeruginosa* was detected in the control group.

**Conclusions:**

This study, the first of its kind from Ireland where CF prevalence is highest, does not provide unequivocal support for prophylactic use of palivizumab in CF patients under 2 years. Despite being derived from a small sample size, based on these data and complementary clinical observation, we have discontinued such prophylaxis. However, should reported incidence of RSV-related hospitalisation increase, there is scientific plausibility for appropriately powered, randomised, controlled trials of palivizumab.

## Correspondence

Dear Editor,

Respiratory syncytial virus (RSV) is the most commonly attributed cause of lower respiratory tract infections in young infants [1]. As RSV is recognised as affecting almost 100 % of children by 24 months, the use of anti-RSV prophylaxis, in the form of humanised monoclonal antibody palivizumab, has become ubiquitous due to its safety and efficacy profile, especially in the context of preventing hospitalisation of high-risk children, such as those born pre-maturely [1, 2]. Globally, Ireland has the highest prevalence of cystic fibrosis (CF) (2.98 per 10,000 population) [3], a condition associated with increased susceptibility to both bacterial and viral respiratory infection. Despite this, there is no previous report of palivizumab’s prophylactic efficacy in a cohort of Irish CF patient under 24 months.

In the context of paediatric patients, such as those with CF at risk of RSV-related hospitalization, there has been considerable expert discussion regarding effectiveness of palivizumab use, which can be summarized as consensus that insufficient data exist to concretely support the economics, safety and tolerability of its use in this setting [4–6]. Of the extant studies, respondents to a United Kingdom-based survey would prescribe palivizumab if funding were not an issue [7] and, although a large number of CF physicians in North America have prescribed palivizumab to CF infants in their first year of life, only half consider this to be standard care [8]. RSV has been identified as a main cause of hospitalisation in cystic fibrosis (CF) infants, however, hospitalisation was most commonly associated with concomitant bacterial infection and the most severe symptoms of lower airway inflammation were seen in those patients co-infected with RSV and *Pseudomonas aeruginosa* [9]. Indeed, RSV has been proven to enhance *P. aeruginosa* infection of respiratory epithelial cells, suggesting a role for viral-bacterial interactions in exacerbations of CF lung disease [10]. To the best of our knowledge, only one study has reported the effects of palivizumab on *P. aeruginosa* colonization, with a lack of demonstrable efficacy over placebo [11].

Here, with ethical approval, we performed an audit at University Hospital Limerick (Ireland), a tertiary referral centre, to document all children with CF, <24 months old, who received palivizumab during their first year of life (once a month for up to 7 months), over a 5-year period. We compared the data with those of all eligible children with cystic fibrosis for the prior 5 year period who had not received the product. In both cohorts, assessment included: respiratory related hospitalisation; differential diagnosis; nasopharyngeal aspirate; and (due to the association between palivizumab and subsequent *Pseudomonas* infection) colonisation of the lower airways by *P. aeruginosa* at initiation of palivizumab prophylaxis or in the subsequent 12 months. Data are presented as mean ± standard error. Fisher’s exact test was used to compare hospitalisation rates of both groups.

In summary, 19 patients received palivizumab (mean doses: 5.2 ± 0.37) and met the criteria for inclusion in the study group, while 30 CF infants who attended UHL from 2004 to 2009, did not receive palivizumab and constituted the control group. Ten patients (53 %) who received prophylactic palivizumab were hospitalised (mean age at admission: 3.1 ± 0.98 months), with 3 of those cases confirmed using molecular techniques as relating to RSV. Mean age at time of first palivizumab treatment was 5.5 ± 0.74 months. *P. aeruginosa* was isolated from one of those children the following year. No children from the control group were hospitalised (*p* < 0.0001), and no *P. aeruginosa* detected.

These results complement previous reports of prophylactic anti-RSV strategies for children, but add considerably to existing knowledge in detailing outcomes from a geographic region in which prophylaxis could have been especially valuable for paediatric CF patients had a benefit been evident. We observed a low rate of RSV-related hospitalisations overall, in addition to a small sample size, similar to elsewhere, that impaired ability to draw statistically relevant conclusions. However, due to economic constraints and the lack of supportive clinical evidence, palivizumab prophylaxis was discontinued. Our study was limited by not assessing the impact of palivizumab intervention in RSV season only and, furthermore, our results do not address other aspects of palivizumab use or potential benefit, including: its role in infants diagnosed with CF by newborn screening; the possibility that changes to timing of palivizumab administration (when to begin and duration of spacing) may improve performance; similarly, whether increased dosage could enhance outcomes; use therapeutically; emergence of resistance; or variation of antigenic presentation affecting efficacy. However, previous reports on the use of palivizumab in RSV-hospitalisations have demonstrated positive effects [12–14]. Therefore, in summary, we believe that, should reported incidence of RSV-related hospitalisation increase, there is scientific plausibility for appropriately powered, randomised, controlled trials of palivizumab.
